# Supplementary material for: Searching the Optimal Folding Routes of a Complex Lasso Protein
Source: Biophys J. 2019 Jun 7;117(2):214–28. doi: 10.1016/j.bpj.2019.05.025 (PMC6700606; doi:10.1016/j.bpj.2019.05.025)
Supplement: Document S1. Supporting Materials and Methods, Figs. S1–S3, and Table S1 [file mmc1.pdf]

Biophysical Journal, Volume *117*

Supplemental Information

Searching the Optimal Folding Routes of a Complex Lasso Protein

Claudio Perego and Raffaello Potestio

Searching the optimal routes to the folding of a Complex Lasso protein: Supplementary Material

Claudio Perego[1] and Raffaello Potestio[2,3]

[1]Polymer Theory Department, Max Planck Institute for Polymer Research, Mainz, Germany
[2]Department of Physics, University of Trento, Trento, Italy
[3]INFN-TIFPA, Trento Institute for Fundamental Physics and Applications, Trento, Italy

S1 Generation of Hybrid Force-fields

In this section we describe the protocol used to generate the coefficients of the hybrid force-fields in the MFFO method. As mentioned in the main submission, after each optimization step, the set of new candidates $\{K'_k\}_{k=1}^{N_K}$ is composed by the winners, namely the $N_{\rm win}$ that had the best ranking in the previous step, and by $N_K - N_{\rm win}$ hybrid force-fields. The latter are generated via a crossover operation, or recombination, that mixes the $k_i^{\rm ang}$ coming from selected "parent" force-fields, mimicking the chromosomal crossover in biology. The parent force-fields are composed by the winners and by $N_{\rm low}$ low-fit force-fields, introduced to maintain variability in the population. In our calculatons we have generated the low-fit forcefields by randomly picking the $k_i^{\rm ang}$'s from a uniform distribution ranging between $k_{\rm min}$ and $k_{\rm max}$.

Once the parent set is defined the crossover is performed in the following way. As shown in Fig. 12 of the manuscript, crossover points along the backbone are defined, at which the angular coefficients of the parent force-fields are divided in subsets. In our calculations we have defined two crossover points, between residues 42 and 43 and between residues 84 and 85, that is:

$$K = \{k_1^{\rm bend}, \ldots, k_{119}^{\rm bend}, k_1^{\rm tor}, \ldots, k_{118}^{\rm tor}\} = \{K_1, K_2, K_3\}, \tag{S1}$$

where the subsets contained both bending and torsion k's:

$$\begin{aligned} K_1 &= \{k_1^{\rm bend}, \ldots, k_{42}^{\rm bend}, k_1^{\rm tor}, \ldots, k_{42}^{\rm tor}\}, & \text{(S2)} \\ K_2 &= \{k_{43}^{\rm bend}, \ldots, k_{84}^{\rm bend}, k_{43}^{\rm tor}, \ldots, k_{84}^{\rm tor}\}, & \text{(S3)} \\ K_3 &= \{k_{85}^{\rm bend}, \ldots, k_{119}^{\rm bend}, k_{85}^{\rm tor}, \ldots, k_{118}^{\rm tor}\}. & \text{(S4)} \end{aligned}$$

Subsets coming from different parents were recombined at the crossover points to generate the hybrid candidates, formally:

$$H = \{K_1^i, K_2^j, K_3^k\}, \tag{S5}$$

in which i, j and k, the indexes of the original parent force-field, were randomly picked among all possible combinations (with no repetition).

S2 Geometry of Topological Variables

In this section we discuss the structure reduction operated to compute the topological variables defined in the Methods section of the manuscript. We consider the 1-residue-to-1-bead CG representation of

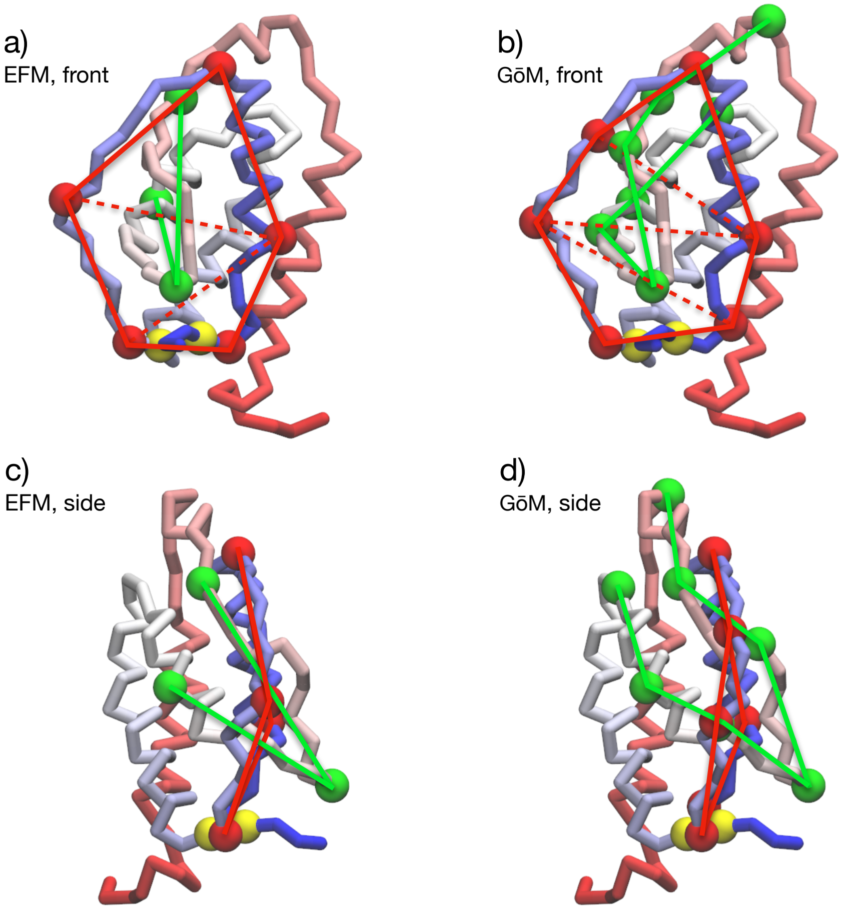

Figure S1: **CG representation of the reduced structures employed for the calculation of L and G.** The loop residues l' are highlighted as red circles connected by red lines, while the threading hairpin residues t' are highlighted as green circles connected by green lines. The red dashed lines indicate the triangulation of the loop surface. The structure reduction adopted for the analysis of EFM trajectories is indicated in a) (front view) and c) (side view), while that employed for the GōM trajectories is indicated in b) (front view) and d) (side view). VMD was employed for the protein visualization[1].

the 2GMF protein, adopted in both the EFM and Gō model simulations presented in the manuscript. In this model the protein is described as a polymer chain of 121 monomers, that we label via the index $i = 1, \ldots, 121$ (the first 3 residues are not resolved in the PDB, the real sequence index of the residues is therefore $j = i + 3$). This CG picture of the protein is displayed in Fig. S1.

The first step required to compute the topological variables is to define $l_1, \ldots, l_{N_l}$, namely the indexes of the covalent loop monomers, and $t_1, \ldots, t_{N_t}$, namely the indexes of the threading hairpin monomers. The covalent loop of 2GMF is formed by the b_1 cysteine bridge, connecting the monomers 85 and 118, highlighted in figure by yellow spheres, we have thus set $l = 85, \ldots, 118$. In the native fold, the covalent loop is pierced by an hairpin formed by residues from $i = 40$ to $i = 50$. In order to include possible fluctuations of the structure we considered a larger set of residues defined by $t = 30, \ldots, 64$. The next step is the definition of the reduced loop and hairpin indexes, $l'_1, \ldots, l'_{M_l}$ and $t'_1, \ldots, t'_{M_t}$ respectively.

As mentioned in Results section of the manuscript, we have operated two different choices for the reduction, depending if the trajectory was produced via EFM or GōM simulation. In the first case we

Model	Env.	P_f	Δ
HM	Red.	0.55	0.0005
	Ox.	0.17	-0.0010
OM	Red.	0.96	0.0024
	Ox.	0.95	0.0044
GōM ($T = 0.7$)	Red.	0.60	0.0005
	Ox.	0.55	0.0010
GōM ($T = 1.1$)	Red.	0.19	-0.440
	Ox.	0.16	-0.112

Table S1: **Probability of folding computed via Q-criterion** Folding probability P_f^Q for each of the considered models, in reducing and oxidizing conditions. The probabilities are estimated as frequency of occurrence over 2048 trajectories of length $\tau_{\text{run}} = 1.5 \times 10^4$, using the Q-criterion defined in the text. Next to each probability we indicate $\Delta = P_f^Q - P_f$, namely the probability difference between the results of the Q-criterion and the RMSD+L-criterion.

have represented the loop by residues $l' = 86, 92, 99, 112, 117$ and the hairpin by residues $t' = 37, 47, 57$, as depicted in Figs S1a) and c). The $M_l - 2 = 3$ triangles spanning the loop surface are also indicated in Fig. S1a. These indexes were used for computing the lasso variable L, while for the Gauss linking number we also added the cysteine residues 85 and 118 to the definition of the loop.

In the GōM case the dihedral stiffness is on average lower than in EFM ($k_1^{\text{tor}} = 1$ and $k_3^{\text{tor}} = 0.5$), and the temperature of interest is larger ($T = 0.1$ in EFM runs while $T = 0.7$ or 1.1 in GōM runs). For this reason the secondary structures are less rigid, and we needed to include more monomers in the definition of the topology. We have represented the loop by residues $l' = 86, 91, 95, 99, 112, 116$ and the hairpin by residues $t' = 30, 37, 41, 47, 51, 57, 64$, shown in Figs S1b) and d). The $M_l - 2 = 4$ triangles spanning the loop surface are shown in Fig. S1b.

S3 Folding Criterion

In our analysis of the folding trajectories (see main text), we have determined the successful foldings using a criterion, which we here name "RMSD+L-criterion", that imposes a maximum RMSD of 0.9 and a minimum L of 0.9 to the conformations belonging to the native state. In this section we compare the results obtained with the RMSD+L-criterion to those deriving from the use of a more standard procedure, based on the fraction of native contacts Q, which we name "Q-criterion". According to the Q-criterion, widely used in the framework of Gō models, the folded state of the protein is reached when all or almost all native contacts are formed simultaneously. The fraction of formed contacts Q is defined as:

$$Q = \frac{1}{N_{\text{c}}} \sum_{i=1}^{N_{\text{c}}} \theta \left(\gamma \mathbf{r}_i^0 - \mathbf{r}_i \right) \tag{S6}$$

where N_c is the number of native contacts, θ is the Heaviside step function, $\mathbf{r}_i$ and $\mathbf{r}_i^0$ are respectively the distance and native distance between the residues of the i-th contact, and γ is a tolerance factor that defines at which distance a contact is established. Following Ref. 2 we have classified as successfully folded all those conformations that exhibit a fraction of contacts larger or equal to $\bar{Q} = 0.97$. We have considered as native contacts all those residues with $\mathbf{r}_i^0 < 2$, separated in sequence by at least three beads. Another crucial parameter for the definition of the criteria is γ, that sets the tolerance in declaring that a contact is established. Again, following Ref. 2, we use $\gamma = 1.2$ for the Gō model, which features a 12-10 LJ potential, while for the EFM, which lacks contact potentials we adopt a larger tolerance setting $\gamma = 1.5$.

The results of using the Q criterion are reported in Tab. S1, showing the difference Δ of success rate with respect to the results of the RMSD+L-criterion, indicated in Tab. 1 of the main text. It can be appreciated that the two criteria show very similar results in the EFM simulations, as well as in the Gō-model runs at $T = 0.7$. The small differences, of the order of few per-thousand, confirm the validity of both criteria in assessing the success of the folding with the employed models, under the considered conditions. Since, in these cases, the outcome differs only in very few trajectories, we could verify the folding of these runs by visual inspection. Overall, we have found the criterion RMSD+L to be more accurate in distinguishing the folded state.

In the remaining cases, that is the Gō-model at $T = 1.1$, we observe a large discrepancy between the two criteria, with the Q-criterion detecting much lower success rates than the ones observed with the RMSD+L criterion. By inspecting exemplars of trajectories in which the two criteria are discordant, we have always observed the establishment of the correct lasso-topology, despite the larger fluctuations entailed by the higher temperature. In this sense the RMSD+L criterion, by detecting the folded state in all the discordant cases, turned out to be more suitable for the presented analysis. Nonetheless, we underline that the accuracy of the Q-criterion can be strongly improved in this $T = 1.1$ by a small change of the tolerance γ. Indeed, by setting $\gamma = 1.25$ we could obtain agreement between the two criteria, with a difference $\Delta = 0.0024$ in the reduced case and no difference ($\Delta = 0$) in the oxidized case.

S4 Optimized Forcefields

In this section we report the coefficients of the optimized forcefields adopted for the EFM study presented in the main manuscript. In Fig. S2 the bending (A) and torsion (B) stiffnesses for the OM and HM model are displayed. The latter are equal to the average values of the optimized bending and torsion coefficients.

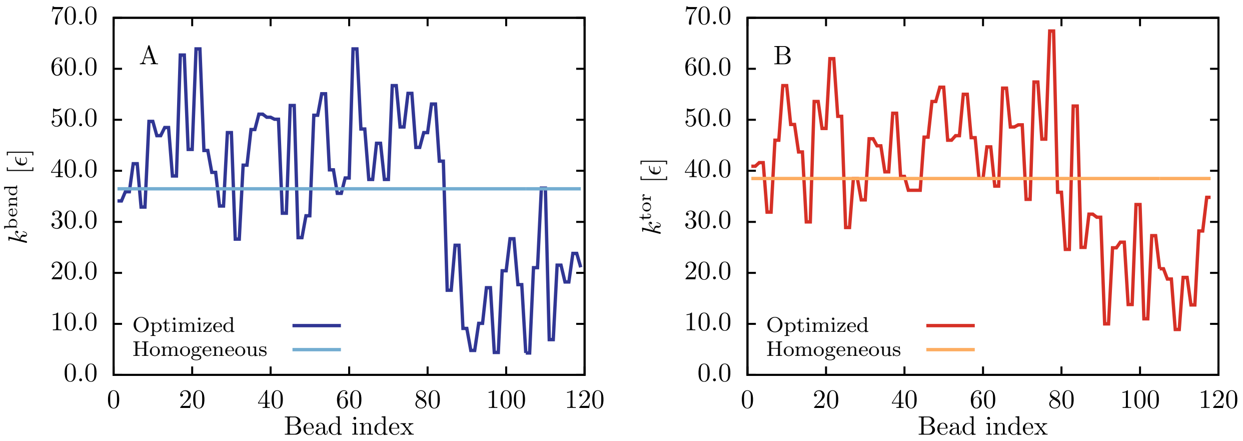

Figure S2: **Angular coefficients of the optimized and homogeneous models, OM and HM**. Panel A and B display the bending and torsion stiffness, respectively.

S5 Gō Model Temperature Study

The GōM used in the present paper was proposed by Clementi et al. to investigate the folding of small globular proteins[3], as mentioned in the manuscript we have generated the model by means of the SMOG web server (http://smog-server.org)[4,5]. Before comparing the results of the GōM with the EFM simulations we have performed a study on the folding propensity of the GōM at different

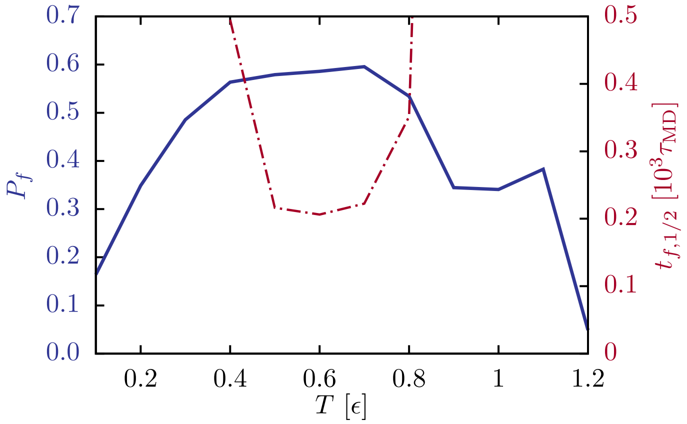

Figure S3: **Temperature range of fastest folding for the GōM.** Folding probability P_f (blue, solid line, left y-axis) and median folding time $t_{f,1/2}$ (red, dot-dashed line, right y-axis) of the GōM at different temperatures.

temperatures, in order to find the range of optimal folding kinetics, at which the fastest folding occurs [6]. We have simulated the folding of 2GMF under reductive conditions, over a range of temperatures from $T = 0.1$ to $T = 1.2$, with spacing $\Delta T = 0.1$. For each value of T we have performed a set of 1024 GōM folding runs of length $\tau_{\mathrm{run}} = 3500$ and estimated the folding probability P_f and time t_f. Our estimate of P_f is equal to the frequency of folding events along the trajectories, it is therefore dependent on the simulation length. Our choice of τ_{run} is justified by the measured median folding times $t_{f,1/2}$, displayed in Fig. S3, where the estimated P_f at different temperatures is displayed as well. Based on these results we have selected to study the GōM model at $T = 0.7$.

References

[1] Humphrey W, Dalke A and Schulten K 1996 *J Mol Graphics* **14** 33–38

[2] Wołek K and Cieplak M 2016 *J Chem Phys* **144** 185102

[3] Clementi C, Nymeyer H and Onuchic J N 2000 *J Mol Biol* **298** 937 – 953 ISSN 0022-2836

[4] Noel J K, Levi M, Raghunathan M, Lammert H, Hayes R L, Onuchic J N and Whitford P C 2016 *PLOS Comput Biol* **12** 1–14

[5] Noel J K, Whitford P C and Onuchic J N 2012 *J Phys Chem B* **116** 8692–8702

[6] Sułkowska J I and Cieplak M 2008 *Biophys J* **95** 3174 – 3191 ISSN 0006-3495
